# Genetic Analysis of a Large-Scale *Phaeocystis globosa* Bloom Offshore Qingdao, China

**DOI:** 10.3390/microorganisms10091723

**Published:** 2022-08-26

**Authors:** Huiyin Song, Yiqi Wang, Xiangxiang Ding, Nansheng Chen

**Affiliations:** 1 College of Life Sciences, Jianghan University, Wuhan 430056, China; 2 CAS Key Laboratory of Marine Ecology and Environmental Sciences, Institute of Oceanology, Chinese Academy of Sciences, Qingdao 266071, China; 3 Laboratory of Marine Ecology and Environmental Science, Qingdao National Laboratory for Marine Science and Technology, Qingdao 266200, China; 4 Center for Ocean Mega-Science, Chinese Academy of Sciences, Qingdao 266071, China; 5 University of Chinese Academy of Sciences, Beijing 100049, China; 6 Department of Molecular Biology and Biochemistry, Simon Fraser University, 8888 University Drive, Burnaby, BC V5A 1S6, Canada

**Keywords:** harmful algal bloom (HAB), *P. globosa* genetic diversity, *pgcp1*, *cox1*, offshore Qingdao

## Abstract

A sudden large-scale bloom event of the haptophyte *Phaeocystis globosa* that lasted over one month in the winter of 2021 was observed offshore Qingdao, China. This *P. globosa* bloom event was unusual as it was the first *P. globosa* bloom recorded in Qingdao offshore. Furthermore, the temperature at which this event occurred was much lower than that of previous *P. globosa* blooms in China. We hypothesize that the *P. globosa* strains that drove the development of this bloom offshore Qingdao were genetically unique and have a competitive advantage in the environmental conditions. To test this hypothesis, we analyzed *P. globosa* genetic diversity and the temporal dynamics of the bloom, using the high-resolution molecular markers *pgcp1* and *cox1* that we developed recently. The analysis revealed that the genetic compositions of *P. globosa* offshore Qingdao were rather limited, containing two dominant genotypes and other rare genotypes with low abundance, representing a small portion of the genetic diversities identified in coastal waters in China, and were rather different from the *P. globosa* genotypes outside of the Jiaozhou Bay before the *P. globosa* bloom in the winter of 2021. This suggested only certain strains contribute to the development of blooms under certain environmental conditions. The genetic composition may indicate the unusual timing and scale of this *P. globosa* event.

## 1. Introduction

The haptophyte *Phaeocystis globosa* blooms are a growing ecological problem in many coastal ecosystems. *P. globosa* blooms can not only pose a negative impact on other co-existing organisms in the ocean [1,2,3,4], but also produce intense mucilage that block the intake of nuclear power cooling systems and threaten the safety of nuclear power infrastructures [5].

*Phaeocystis globosa* blooms have been observed in the central Arabian Sea during the summer monsoon period (July–August, 1996) [6]; in the Dutch Wadden Sea with a high spring peak [7]; in the coastal waters of the North Sea during spring, indirectly determined by the combined effect of the North Atlantic oscillation (NAO) and freshwater and continental nitrate [8]; in the south central coastal waters of Vietnam, frequently associated with upwelling events [9]; and on the South Atlantic Bight continental shelf during the summer, also associated with upwelling events [10]. In China, since the first record of *P. globosa* blooms in 1997–1998 in the South China Sea [2,11], *P. globosa* blooms have been frequently recorded in almost all coastal regions [11,12], including the South China Sea [13] and the Bohai Sea [12]. Notably, *P. globosa* blooms have been rare in the Yellow Sea regions [13,14] and no *P. globosa* blooms have been previously recorded offshore Qingdao. 

Environmental conditions in which *P. globosa* blooms occur were rather variable. Temperatures at which *P. globosa* blooms occurred in the eastern English Channel [15] ranged from 6.1 °C to 17.5 °C, while temperatures at which *P. globosa* blooms occurred in Vietnamese waters were much higher, ranging from 24 °C to 29.5 °C [9,16]. In China, *P. globosa* blooms usually occurred when water temperatures were about 15–30 °C [12,13,14]. Consistent with the differences in temperatures at which *P. globosa* blooms occur, *P. globosa* blooms in different ocean regions were also characterized by different colony sizes. While *P. globosa* colony sizes were generally small (8–9 mm) in European coastal waters [17,18,19], *P. globosa* usually form giant colonies in coastal waters in China and in Vietnam coastal regions (up to 3.0 cm) [13]. Such dramatic differences in temperatures at which *P. globosa* blooms occur and in colony sizes during bloom suggest that *P. globosa* may has high genetic diversity, which was confirmed by genetic analysis of *P. globosa* strains isolated in different coastal waters using high resolution molecular markers *pgcp1* and *cox1* [20,21]. 

Offshore Qingdao, which extends from Rizhao City to Qingdao City, is a part of the Yellow Sea. A sudden large-scale (~1500 km^2^) *P. globosa* bloom was observed from offshore Qingdao from ~Nov 20 to ~Dec 31, 2021 for over 40 days. This *P. globosa* event was unusual not only for being first offshore Qingdao, but also for the low water temperature at which the event occurred, which was 6.6–12.0 °C. The low instance of *P. globosa* blooms offshore Qingdao has been hypothesized to be due to the frequent occurrences of green tide in the ocean region [14]. Interestingly, the exceptional *P. globosa* bloom event that occurred in the winter of 2021 coincided with the strongest green tide developed in the Yellow Sea in the same year (Bulletin of China Marine Disaster, 2021).

Because of the occurrence of the *P. globosa* bloom offshore Qingdao in the Yellow Sea at low temperature (6.6–12.0 °C), we hypothesized that the bloom was caused by *P. globosa* strains with unique genetic background. To test this hypothesis, we characterized the *P. globosa* genetic diversity and its temporal variation in the *P. globosa* bloom from offshore Qingdao, China, using the molecular markers *pgcp1* and *cox1* that were capable of distinguishing *P. globosa* strains with high resolution. These molecular markers were used because common molecular markers including 18S rDNA and ITS were ineffective in distinguishing *P. globosa* genetic diversity [17,21,22]. These analyses revealed that certain *P. globosa* strains were associated with the development of *P. globosa* blooms offshore Qingdao, China, in the winter of 2021.

## 2. Materials and Methods

### 2.1. Field Sampling

**Figure 1:**
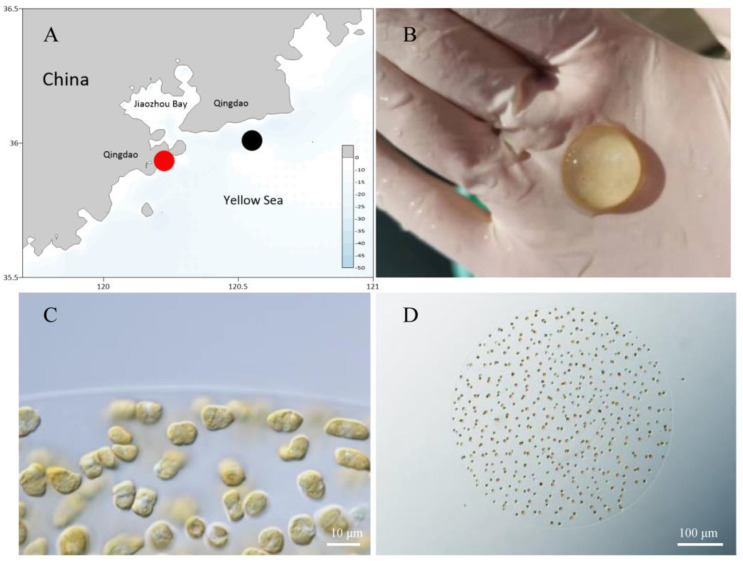
Geographical locations of sampling sites and morphological characteristics of *P. globosa* colonies. (**A**) Geographical locations of the sampling sites for field samples: red circle: the sampling site in this study; black circle: the sampling site at which *P. globosa* was sampled outside of the Jiaozhou Bay in January 2019. (**B**) A *P. globosa* colony. (**C**) A portion of a *P. globosa* colony under a light microscope, with *P. globosa* cells observable. (**D**) Morphological characteristics of a small-sized *P. globosa* colony under a light microscope.

In order to analyze *P. globosa* genetic diversity and the temporal variation of *P. globosa* strains during the *P. globosa* bloom that occurred in the winter of 2021 offshore Qingdao, we sampled the bloom at its peak and during the declining phase. Seven field samples were collected approximately weekly on Dec 3, Dec 8, Dec 11, Dec 16, Dec 21, Dec 26, and Dec 31, 2021 from the same location, Lu Haifeng Marine Ranch, the coordinates of which are 120.24° E, 35.93° N (red circle in Figure 1A). The colonies were visible to the naked eye throughout the sampling period. The colonies turned white and no cell morphology was recognizable on the last sampling day (Dec 31, 2021). On each sampling day, water temperature and salinity were measured in situ by the authors. Three replicates of 1L water samples were collected from the surface for DNA extraction. Water samples were quickly brought back to the laboratory and filtered using 0.20 μm polycarbonate membranes (Millipore, Billerica, MA, USA). The polycarbonate membranes were then stored in liquid nitrogen. The chlorophyll-a (Chl *a*) determinations were filtered through 0.7 µm glass microfiber filters (Whatman, Maidstone, UK, GAT No. 1825-025) and filters were preserved at −20 °C in darkness before further laboratory processing. pH was determined using a Mettler Toledo Seven Compact pH meter (Billerica, MA, USA); PO_4_^3−^, NO_3_^−^, NO_2_^−^, NH_4_^+^, SiO_3_^2−^ and chlorophyll-a data were measured by Analysis and Testing Center, Institute of Oceanology, Chinese Academy of Sciences, using methods that were specified in the Chinese National Standards (GB/T12763.4-2007 and GB/T12763.6-2007) for data quality assurance.

### 2.2. Morphological Observation

Morphological features of *P. globosa* colonies were observed onsite (Figure 1B), while detailed morphological features were observed in lab using a light microscope (ZEISS Axio Imager Z2) (Figure 1C) and a stereo microscope (ZEISS Stemi 305) (Figure 1D).

### 2.3. DNA Preparation

Genomic DNA was extracted using the HP Plant DNA Mini Kit (Omega Bio-tek, Inc., Norcross, GA, USA). DNA concentrations and quality were determined using a NanoDrop 2000 spectrophotometer (Labtech International Ltd., Uckfield, UK) and were resolved using agarose gel electrophoresis.

### 2.4. PCR Amplification, Cloned and Sanger Sequencing

To analyze genetic diversity of *P. globosa* in the field samples, the DNA extracted from each sample was PCR amplified using primers targeting molecular markers *pgcp1* and *cox1* using PCR primers described previously [21] and a high-fidelity DNA polymerase (PrimeSTAR Max DNA Polymerase, Takara Bio Inc., Kusatsu, Japan). PCR products were purified using the GE0101–200 Kit (TsingKe, Beijing, China), and cloned using the pClone007 Versatile Simple Vector Kit (TsingKe, Beijing, China), followed by Sanger sequencing. A total of 140 *pgcp1* sequences and 170 *cox1* sequences were obtained. Combined with the newly obtained sequences in this study and previously obtained from other sea areas, we used CD-HIT-EST clusters [23] to calculate 100% identical repeated sequences, retaining only one representative, and then obtained the non-redundant sequences. As a result, a total of 19 *pgcp1* genotypes and a total of 18 *cox1* genotypes were found in these *P. globosa* samples.

### 2.5. Phylogenetic Analyses

Sequences of each molecular marker were aligned with MAFFT [24] and adjusted manually with MEGA 7.0 [25]. Ambiguous sites of the sequence fragments were removed from further analyses. Neighbor-joining (NJ) trees were inferred using MEGA 7.0, and the support for nodes was assessed by performing 1000 bootstrap replicates.

### 2.6. Network Construction Using TCS

We also constructed a network that displayed phylogenetic relationships among haplotypes for *pgcp1* and *cox1* genotypes identified in this bloom using TCS construction package POPART 1.7 [26]. The sequences with indels were excluded from TCS network analysis.

### 2.7. Correlation Analysis between Genotypes and Environmental Factors

Redundancy analysis (RDA) was conducted with online website (http://www.cloud.biomicroclass.com/CloudPlatform/SoftPage/CCA) accessed on 3 August 2022, based on the sequence number of the *P. globosa* genotypes in each sample.

## 3. Results

### 3.1. Description of the First Large-Scale P. globosa Bloom Recorded Offshore Qingdao 

An unexpected large-scale *P. globosa* bloom event was recorded in coastal regions of Qingdao, Shandong Province, China, from ~Nov 20 to ~Dec 31, 2021 for over 40 days, with the water temperature ranging from 6.6–12.0 °C. The bloom covered approximately 1500 km^2^, extending from Rizhao City to the opening of the Jiaozhou Bay in Qingdao City, Shandong Province, China (Figure 1A). *Phaeocystis globosa* colonies had various sizes, with large ones reaching 1.5 cm in diameter (Figure 1B). Individual cells were generally homogenously distributed at the periphery of the colonies, some of which showed shallow cracks. Most of the cells observed in the field colonies were in the two-division state, and the cells were in the rapid division stage, and no flagella were seen (Figure 1C, D), suggesting that the colonies were growing during sampling. The colonies and cells resembled those of *P. globosa* reported previously in other regions of China, including the Zhelin Bay, Guangdong, China [1], and the Beibu Gulf, Guangxi Province, China [19].

### 3.2. Genetic Dissection of P. globosa Bloom Development Using pgcp1

**Figure 2:**
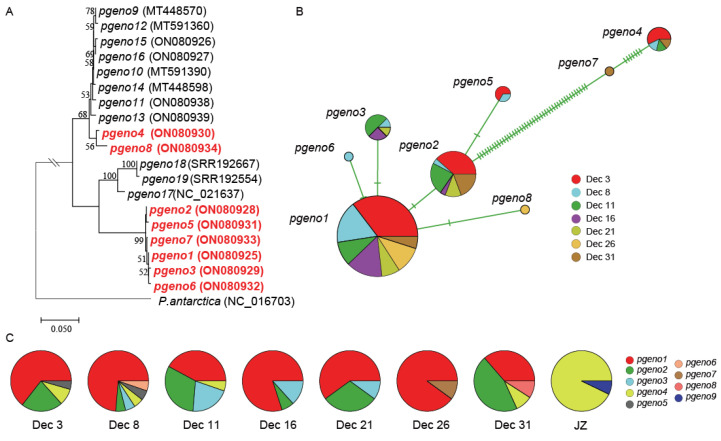
The phylogenetic analysis of *P. globosa*, genetic diversity and temporal dynamics of the *P. globosa* bloom using molecular marker *pgcp1*. (**A**) The phylogenetic analysis based on *pgcp1* non-redundant sequences, and the genotypes found in this bloom are in red. (**B**). A TCS network of the *pgcp1* sequences of *P. globosa* that were obtained from 7 field samples. Each circle represents a different haplotype. The size of each circle is proportional to the number of sequences for each haplotype. Each line connecting two haplotypes indicates nucleotide change regardless of the length, and one bar on the lines represents one base difference. (**C**) Genotype composition of different sampling times. JZ: sample collected outside of the Jiaozhou Bay in January 2019.

To characterize the *P. globosa* genetic composition, a total of 140 *pgcp1* sequences were obtained, containing eight genotypes (*pgeno1*–*pgeno8*) (Table 1, Figure 2A). Combined with the *pgcp1* sequences previously obtained from other sea areas, a total of 19 *pgcp1* genotypes were identified (Table 1, Figure 2A). Phylogenetic analysis revealed that these eight genotypes amplified in Qingdao field samples formed two tightly linked clusters (Figure 2A); one cluster included two genotypes, *pgeno4* and *pgeno8*, while another cluster included six genotypes, *pgeno1*, *pgeno2*, *pgeno3*, *pgeno5*, *pgeno6*, and *pgeno7*. These eight genotypes showed a remarkable difference in abundance, among which *pgeno1* (59% of all obtained sequences) and *pgeno2* (19% of all obtained sequences) dominated the *P. globosa* blooms in Qingdao water (Figure 2B).

Comparative analysis of the molecular marker *pgcp1* sequences obtained in this study and in previous studies [20] uncovered that *pgeno1* was also identified in *P. globosa* strains isolated from the Beibu Gulf and in the coastal regions of Zhangpu, Fujian Province, China (Table 1); *pgeno2* was also identified in the Beibu Gulf, Lianyungang, the Daya Bay, and in the coastal regions of Zhangpu, Fujian Province, China; *pgeno3* was also identified in the Beibu Gulf. In contrast, *pgeno4*–*pgeno8* genotypes were only identified in this study in the coastal regions of Qingdao. *pgeno9*, which was previously identified outside of the Jiaozhou Bay (but not in this bloom), was also identified in coastal regions of Thailand, the South China Sea, and samples collected in the South Pacific Ocean.

While *pgeno1* and *pgeno2* were the dominant genotypes, compositions of *P. globosa* genotypes changed only slightly during the bloom development from Dec 3 to Dec 31 (Figure 2C). Notably, *pgeno8* was only found during the fading stage of the *P. globosa* blooms. Interestingly, *P. globosa* genotype compositions during the bloom showed a remarkable difference from the *P. globosa* genotypes reported previously outside of the Jiaozhou Bay in January 2019 (Figure 2C) [20].

### 3.3. Phaeocystis globosa Genotype Composition during Bloom Development Revealed Using cox1

**Figure 3:**
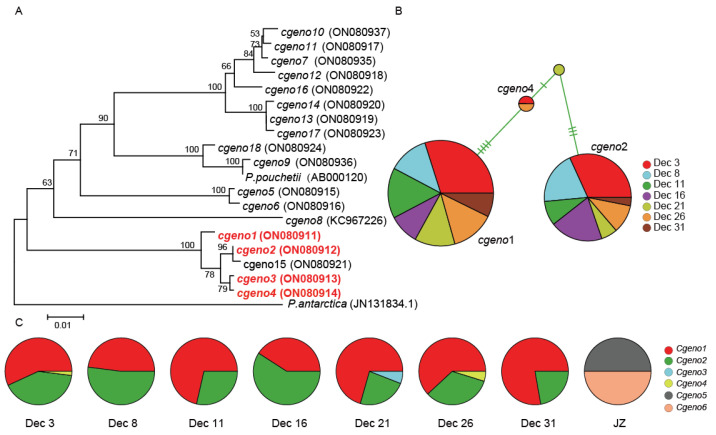
The phylogenetic analysis of *P. globosa*, genetic diversity and temporal dynamics of the *P. globosa* bloom using molecular marker *cox1*. (**A**) The phylogenetic analysis based on *cox1* non-redundant sequences, and the genotypes found in this bloom are in red. (**B**) A TCS network of the *cox1* sequences of *P. globosa* that were obtained from 7 field samples. Each circle represents a different haplotype. The size of each circle is proportional to the number of sequences for each haplotype. Each line connecting two haplotypes indicates nucleotide change regardless of the length, and one bar on the lines represents one base difference. (**C**) Genotype composition at different sampling times. JZ: sample collected outside of the Jiaozhou Bay in January 2019.

We also examined the *P. globosa* genotype compositions during *P. globosa* bloom development using another molecular marker, *cox1*, which was developed based on the mitochondrial genome [21]. A total of 170 *cox1* sequences were obtained, containing four genotypes (*cgeno1*–*cgeno4*) (Table 2, Figure 3A). Combined with *cox1* sequences previously obtained, a total of 18 *cox1* genotypes were found (Table 2, Figure 3A). Phylogenetic analysis revealed that these four genotypes amplified in Qingdao field samples formed three clusters (Figure 3A), including *cgeno1*, *cgeno2*, and *cgeno3*–*4*, respectively. These four genotypes showed a remarkable difference in abundance, among which *cgeno1* (57% of all obtained sequences) and *cgeno2* (39% of all obtained sequences) dominated the *P. globosa* blooms in Qingdao water (Figure 3B).

Comparative analysis of the molecular marker *cox1* sequences obtained in this study and in previous studies [21] uncovered that *cgeno1* was also identified in *P. globosa* strains isolated from the Beibu Gulf (Table 2); *cgeno2* was also identified in the Beibu Gulf, Lianyungang, Daya Bay, and Fujian Sea area; *cgeno3* was also identified in the Beibu Gulf. In contrast, *cgeno4* was only identified in the coastal regions of Qingdao. *Cgeno5* and *cgeno6* were only previously identified outside of the Jiaozhou Bay (but not in this bloom).

While *cgeno1* and *cgeno2* were the dominant genotypes, compositions of *P. globosa* genotypes changed slightly during the bloom development from Dec 3 to Dec 31 (Figure 3C). Interestingly, *P. globosa* genotype compositions during the bloom showed a remarkable difference from the *P. globosa* genotypes reported previously outside of the Jiaozhou Bay in January 2019 (Figure 3C) [21].

### 3.4. Environmental Factors and Their Influence on P. globosa Genotype Composition

**Figure 4:**
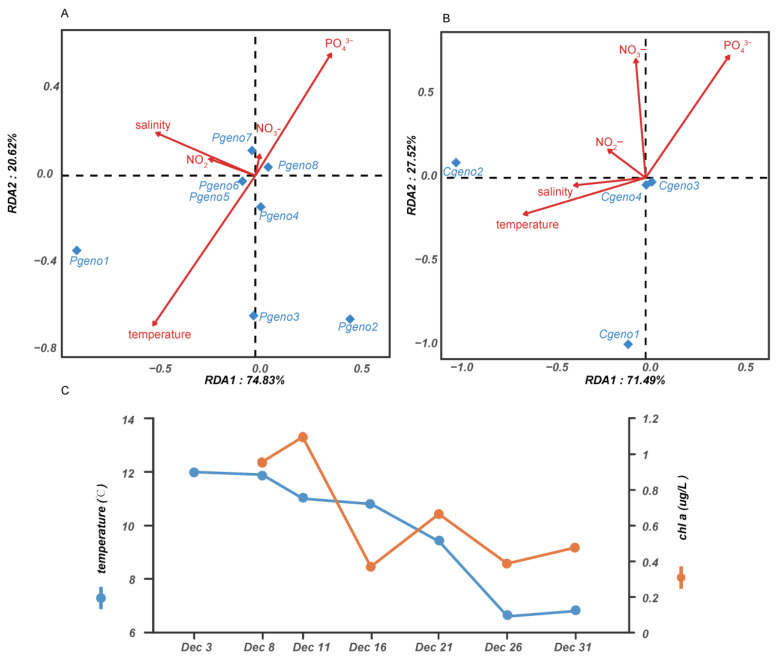
Redundancy analysis (RDA) of the environmental factors. (**A**) The *pgcp1* genotypes. (**B**) The *cox1* genotypes. Blue tetrapods indicate genotypes. (**C**) Measurements of temperature and Chl *a* concentration during the sampling period.

The ranges of temperature, salinity, pH, PO_4_^3−^, NO_3_^−^, NO_2_^−^, NH_4_^+^, SiO_3_^2−^, and chlorophyll-a at the sampling sites were 6.6 °C–12.0 °C (temperature), 27.0–29.6 (salinity), 8.03–8.77 (pH), 3.78–7.42 ug/L (PO_4_^3−^), 47.96–90.78 ug/L (NO_3_^−^), 2.00–7.47 ug/L (NO_2_^−^), 33.24–161.97 ug/L (NH_4_^+^), 6.82–20.27 ug/L (SiO_3_^2−^) and 0.370–1.095 ug/L (chlorophyll-a), respectively (Table S1). During the peak and late phases of the *P. globosa* bloom, the temperature decreased slightly, and the salinity changed slightly. NO_3_^−^ had the lowest value and NH_4_^+^ had the highest value on Dec 21, 2021. pH decreased slightly in decline phase (Dec 31, 2021). To explore the impact of environmental factors on *P. globosa* genotype composition, the correlation between *P. globosa* genotype composition and environmental factor was calculated (Figure 4). Temperature, salinity and NO_2_^−^ had a substantial positive connection with the dominant genotypes *pgeno1*, *cgeno1* and *cgeno2*. The highest levels of chlorophyll-a were observed in the middle phases of the *P. globosa* bloom (Dec 8, 2021 and Dec 11, 2021).

## 4. Discussion

### 4.1. An Unusual P. globosa Bloom Event Observed Offshore Qingdao, China

The sudden occurrence of a large-scale *P. globosa* bloom offshore Qingdao in the winter of 2021 demonstrated that *P. globosa* blooms can occur in the Yellow Sea and can occur at a temperate that is much lower than temperatures at which previous *P. globosa* blooms have occurred, substantially broadening our understanding of appropriate environmental conditions for *P. globosa* blooms. Previously, *P. globosa* blooms were frequently observed in many ocean regions around the world, and the suitable temperature was generally high, ranging from 6.1 °C to 17.5 °C in the eastern English Channel [15], from 24 °C to 29.5 °C in Vietnamese waters [9,16], from 15 °C to 27 °C in the South China Sea [13], and from ~15 °C to 20.8 °C in the Bohai Sea, China [12]. *Phaeocystis globosa* blooms were rarely observed in the Yellow Sea [14] and they have never been reported offshore Qingdao. We observed a obvious temperature decrease on Dec 26, 2021 (consistent with [27]); then, on the following sampling day (Dec 31, 2021), the colonies turned white and the cells were dying and becoming unrecognizable, suggesting that cooling might be a determining factor for the demise of *P. globosa* blooms. Correlation analysis also confirmed that temperature was positively corrected with the dominant genotypes *pgeno1*, *cgeno1* and *cgeno2*.

### 4.2. Genetic Uniqueness of P. globosa in Bloom Development

Comparative analyses of the genetic composition of the *P. globosa* samples collected offshore Qingdao using high resolution molecular markers *pgcp1* and *cox1* revealed consistently that this bloom consisted of only two dominant genotypes, among a large number of genotypes (Figure 2A and Figure 3A), suggesting that genetic uniqueness might be essential in the *P. globosa* bloom development offshore Qingdao in the winter of 2021. Analysis using *pgcp1* revealed only two dominant genotypes, *pgeno1* and *pgeno2*, while analysis using *cox1* revealed only two primary genotypes, *cgeno1* and *cgeno2*. While other genotypes, such as *cgeno3- cgeno4* and *pgeno6*–*pgeno8*, all had only a minor contribution, suggesting a low proportion of the population. During the entire sampling duration, the relative abundance of different genotypes remained rather stable with minimum changes. Despite the rare occurrences of *P. globosa* blooms in the Yellow Sea, the presence of *P. globosa* has been detected outside of the Jiaozhou Bay using high resolution molecular markers *pgcp1* and *cox1* that we have recently developed [20,21], suggesting that *P. globosa* strains exist in coastal regions with rare *P. globosa* blooms. Notably, *P. globosa* genotypes recorded during this bloom showed a remarkable difference from the *P. globosa* genotypes reported previously outside of the Jiaozhou Bay in January 2019. This suggests that although *P. globosa* genetic diversity is high in coastal regions including offshore Qingdao, only certain strains contribute to the development of blooms under certain environmental conditions.

### 4.3. Source of P. globosa Strains Driving the Bloom Offshore Qingdao, China

The two dominant *P. globosa* genotypes detected in the bloom offshore Qingdao in the winter of 2021 were also found in other ocean areas including the Beibu Gulf, Guangxi Province, offshore Lianyungang, Jiangsu Province, the Daya Bay, Guangdong Province and Fujian province, suggesting the widespread presence of these genotypes, and perhaps all *P. globosa* genotypes, at various relative abundances. Nevertheless, genetic analysis of samples collected outside of the Jiaozhou Bay before the *P. globosa* bloom in the winter of 2021 and samples collected during the bloom were rather different, suggesting that the two dominant *P. globosa* genotypes could be introduced from other ocean regions, or that they were local but at low relative abundance. Regardless of the source of *P. globosa* strains, the occurrence of the *P. globosa* observed during the winter of 2021 offshore Qingdao suggests that these two dominant genotypes may be responsive to environmental factors.

## 5. Conclusions

The observation of the sudden *P. globosa* bloom in the winter of 2021 offshore Qingdao suggests that *P. globosa* blooms can occur in the Yellow Sea, in which *P. globosa* blooms have rarely been reported, during a year with strong green tide, and at temperatures that are much lower than those at which previous *P. globosa* blooms in China occurred. Thus, analysis of this *P. globosa* bloom has broadened our understanding of *P. globosa* bloom development. The identification of only two dominant *P. globosa* genotypes in the *P. globosa* bloom suggests that these genotypes may be responsive to environmental factors including low temperature. Although these two genotypes were also identified previously in other ocean regions, these genotypes may not have contributed to the development of *P. globosa* blooms observed in other ocean regions. Alternatively, these two genotypes may be eurythermal and able to develop *P. globosa* blooms in winter offshore Qingdao, and also develop *P. globosa* blooms at higher temperatures in other ocean regions. How these genotypes differ from other *P. globosa* genotypes in responding to environmental factors needs to be further investigated.

## Figures and Tables

**Table 1:** Geographic origin and GenBank accession of *pgcp1* genotype.

Genotype	Geographic Origin of Water Samples	Geographic Origin of Strains	GenBank Accession or SRA Read Numbers
*pgeno1*	Qingdao, China	Beibu Gulf, China; Fujian, China	ON080925
*pgeno2*	Qingdao, China	Beibu Gulf, China; Lianyungang, China; Daya Bay, China; Fujian, China	ON080928
*pgeno3*	Qingdao, China	Beibu Gulf, China	ON080929
*pgeno4*	Qingdao, China		ON080930
*pgeno5*	Qingdao, China		ON080931
*pgeno6*	Qingdao, China		ON080932
*pgeno7*	Qingdao, China		ON080933
*pgeno8*	Qingdao, China		ON080934
*pgeno9*	Qingdao, China	Thailand; the South China Sea; South Pacific	MT448570
*pgeno10*		Beibu Gulf, China; the South China Sea	MT591390
*pgeno11*		Gulf of Mexico; Ocean North Atlantic; NA	ON080938
*pgeno12*		Beibu Gulf, China	MT591360
*pgeno13*		Ocean North Atlantic	ON080939
*pgeno14*		Viet Nam	MT448598
*pgeno15*		the South China Sea	ON080926
*pgeno16*		the South China Sea	ON080927
*pgeno17*		Ocean North Atlantic; Europe’s North Sea	NC_021637
*pgeno18*	NA	NA	SRR192667.99032.2
*pgeno19*	NA	NA	SRR192554.305016.2

**Table 2:** Geographic origin and GenBank accession of *cox1* genotype.

Genotype	Geographic Origin of Water Samples	Geographic Origin of Strains	GenBank Accession
*cgeno1*	Qingdao, China; Beibu Gulf, China	Beibu Gulf, China	ON080911
*cgeno2*	Qingdao, China; Beibu Gulf, China	Beibu Gulf, China; Lianyungang, China; Daya Bay, China; Fujian, China	ON080912
*cgeno3*	Qingdao, China	Beibu Gulf, China	ON080913
*cgeno4*	Qingdao, China		ON080914
*cgeno5*	Qingdao, China		ON080915
*cgeno6*	Qingdao, China		ON080916
*cgeno7*	Beibu Gulf, China	Beibu Gulf, China; Thailand; Viet Nam; the South China Sea; North Pacific; South Pacific	ON080935
*cgeno8*		Ocean North Atlantic; Europe’s North Sea	KC967226
*cgeno9*		Ocean North Atlantic	ON080936
*cgeno10*		Gulf of Mexico; Ocean North Atlantic; NA	ON080937
*cgeno11*		Viet Nam	ON080917
*cgeno12*		the South China Sea	ON080918
*cgeno13*	Beibu Gulf, China		ON080919
*cgeno14*	Beibu Gulf, China		ON080920
*cgeno15*	Beibu Gulf, China		ON080921
*cgeno16*	Beibu Gulf, China		ON080922
*cgeno17*	Beibu Gulf, China		ON080923
*cgeno18*	Beibu Gulf, China		ON080924

## Data Availability

Sequencing results had been submitted to NCBI under GenBank accession in Table 1 and Table 2.

## Supplementary Materials

The following supporting information can be downloaded at: https://www.mdpi.com/article/10.3390/microorganisms10091723/s1, Table S1: List of all samples and corresponding environmental factors.
