# Supplementary material for: Genetic Analysis of a Large-Scale Phaeocystis globosa Bloom Offshore Qingdao, China
Source: Microorganisms. 2022 Aug 26;10(9):1723. doi: 10.3390/microorganisms10091723 (PMC9500930; doi:10.3390/microorganisms10091723)
Supplement: Supplementary file 1 [file microorganisms-10-01723-s001.zip › microorganisms-1813920-supplementary.pdf]

microorganisms

**Table S1.** List of all samples and corresponding environmental factors.

| Sample Date | Temperature | Salinity | $PO_4^{3-}$ | $NO_3^-$ | $NO_2^-$ | $NH_4^+$ | $SiO_3^{2-}$ | PH | Chlorophyll-A |
|---|---|---|---|---|---|---|---|---|---|
| 03 December 2021 | 12.0 | NA | NA | NA | NA | NA | NA | NA | NA |
| 08 December 2021 | 11.9 | 29.3 | 5.05 | 65.40 | 7.47 | 48.49 | 17.37 | 8.46 | 0.954 |
| 11 December 2021 | 11.0 | 28.2 | 3.78 | 63.74 | 2.29 | 47.26 | 6.82 | 8.77 | 1.095 |
| 16 December 2021 | 10.8 | 28.2 | 5.01 | 86.03 | 4.24 | 61.34 | 19.22 | 8.54 | 0.370 |
| 21 December 2021 | 9.4 | 27.0 | 5.07 | 47.96 | 7.47 | 161.97 | 12.16 | 8.51 | 0.665 |
| 26 December 2021 | 6.6 | 29.6 | 5.33 | 70.27 | 2.00 | 33.24 | 18.12 | 8.49 | 0.388 |
| 31 December 2021 | 6.8 | 28.4 | 7.42 | 90.78 | 3.90 | 50.22 | 20.27 | 8.03 | 0.477 |

NA: Missing data.
